# Distinct mast cell subpopulations within and around lymphatic vessels regulate lymph flow and progression of inflammatory-erosive arthritis in TNF-transgenic mice

**DOI:** 10.3389/fimmu.2023.1275871

**Published:** 2023-12-14

**Authors:** Yue Peng, H. Mark Kenney, Karen L. de Mesy Bentley, Lianping Xing, Christopher T. Ritchlin, Edward M. Schwarz

**Affiliations:** ^1^ Center for Musculoskeletal Research, University of Rochester Medical Center, Rochester, NY, United States; ^2^ Department of Pathology & Laboratory Medicine, University of Rochester Medical Center, Rochester, NY, United States; ^3^ Department of Medicine, Division of Allergy, Immunology, Rheumatology, University of Rochester Medical Center, Rochester, NY, United States

**Keywords:** T32GM007356, F30AG076326, R01AG059775, R01AR069000, R01AR056702, mast cell, lymphatic vessel, inflammatory-erosive arthritis

## Abstract

**Objective:**

Inflammatory-erosive arthritis is exacerbated by dysfunction of joint-draining popliteal lymphatic vessels (PLVs). Synovial mast cells are known to be pro-inflammatory in rheumatoid arthritis (RA). In other settings they have anti-inflammatory and tissue reparative effects. Herein, we elucidate the role of mast cells on PLV function and inflammatory-erosive arthritis in tumor necrosis factor transgenic (TNF-tg) mice that exhibit defects in PLVs commensurate with disease progression.

**Methods:**

Whole mount immunofluorescent microscopy, toluidine blue stained histology, scanning electron microscopy, and *in silico* bioinformatics were performed to phenotype and quantify PLV mast cells. Ankle bone volumes were assessed by μCT, while corresponding histology quantified synovitis and osteoclasts. Near-infrared indocyanine green imaging measured lymphatic clearance as an outcome of PLV draining function. Effects of genetic MC depletion were assessed via comparison of 4.5-month-old WT, TNF-tg, MC deficient *Kit^W-sh/W-sh^* (cKit^-/-^), and TNF-tg x cKit^-/-^ mice. Pharmacological inhibition of mast cells was assessed by treating TNF-tg mice with placebo or cromolyn sodium (3.15mg/kg/day) for 3-weeks.

**Results:**

PLVs are surrounded by MCT^+^/MCPT1^+^/MCPT4^+^ mast cells whose numbers are increased 2.8-fold in TNF-tg mice. The percentage of peri-vascular degranulating mast cells was inversely correlated with ICG clearance. A population of MCT^+^/MCPT1^-^/MCPT4^-^ mast cells were embedded within the PLV structure. *In silico* single-cell RNA-seq (scRNAseq) analyses identified a population of PLV-associated mast cells (marker genes: *Mcpt4, Cma1, Cpa3, Tpsb2, Kit, Fcer1a & Gata2*) with enhanced TGFβ-related signaling that are phenotypically distinct from known MC subsets in the *Mouse Cell Atlas*. cKit^-/-^ mice have greater lymphatic defects than TNF-tg mice with exacerbation of lymphatic dysfunction and inflammatory-erosive arthritis in TNF-tg x cKit^-/-^ vs. TNF-Tg mice. Cromolyn sodium therapy stabilized PLV mast cells, increased TNF-induced bone loss, synovitis, and osteoclasts, and decreased ICG clearance.

**Conclusions:**

Mast cells are required for normal lymphatic function. Genetic ablation and pharmacological inhibition of mast cells exacerbates TNF-induced inflammatory-erosive arthritis with decreased lymphatic clearance. Together, these findings support an inflammatory role of activated/degranulated peri-PLV mast cells during arthritic progression, and a homeostatic role of intra-PLV mast cells, in which loss of the latter dominantly exacerbates arthritis secondary to defects in joint-draining lymphatics, warranting investigation into specific cellular mechanisms.

## Introduction

Rheumatoid arthritis (RA) is a chronic inflammatory joint disease that affects up to 1% of the population worldwide (1). Besides pannus tissue invasion, cartilage degradation and focal bone erosion of joints, RA patients show altered lymphatic function, as evidenced by reduced lymphatic vessel contraction and slower lymphatic clearance rate compared to healthy controls (2–4). Three distinct mechanisms underlie lymphatic dysfunction in murine models of RA. First, loss of lymphatic vessel contractions is observed over the course of progressive arthritis secondary to extensive tissue damage in the setting of chronic inflammation (5). Second, accumulation and translocation of B cells to the sinuses obstructs passive lymphatic flow in joint draining lymph nodes (6, 7). Finally, release of local vasodilators, such as nitric oxide, likely generated by peri-lymphatic inflammatory cells that express inducible-nitric oxide synthase, contribute to reduced lymphatic flow (8, 9).

In RA patients, mast cells are also present throughout the RA synovial sublining, with occasional microanatomic clustering in the pannus near sites of cartilage and bone erosion, and comprise 5% or more of the expanded synovial cell population (10–12). Synovial mast cells produce cytokines (i.e. TNF, IL-1 and IL-6) that mediate monocyte/macrophage recruitment, differentiation, and activation, and serve as targets of RA biologic therapies (13–15). Additionally, some mast cells promote long term effects in joint tissues through the recruitment of inflammatory cells and activation of stromal cells resulting in fibrosis and accelerated angiogenesis (15, 16). The number of accumulated mast cells differs substantially from patient to patient and this variation is associated with the intensity of joint inflammation (17). In fact, the presence of mast cells in early RA synovial tissue correlates with disease severity and supports B cell autoantibody production (18). Thus, evidence from preclinical and clinical studies support both a pro-inflammatory and catabolic role for mast cells in RA (19).

Mast cells have also been described as tissue resident, including lymphatics. The literature describes two subtypes of mast cells: mucosal mast cells that produce tryptase and connective tissue mast cells that produce chymase, tryptase, and carboxypeptidases (20). Mast cells are recognized as potent regulators of lymphatic vessel function based on their anatomical proximity and ability to produce, store, and release various inflammatory and vasoactive mediators (21–23). Mast cells release mediators (e.g., histamine) that impact the contractility of lymphatic vessels (21, 23, 24), which is critical for efficient drainage of joint inflammation. Histamine (H)1 receptors enhance and H2 receptors slow down lymphatic contractions, the dominant effect being an increased contractile activity(24).

The role of mast cell regulation of joint draining lymphatics and their functions in RA remains undefined. To identify the effector cells that control lymphatic vessel contractions efferent to inflamed joints in RA, we performed exploratory whole mount immunofluorescent microscopy (WMIFM) on popliteal lymphatic vessels (PLVs) from tumor necrosis factor-transgenic (TNF-tg) mice with inflammatory-erosive arthritis (25) and their wild-type (WT) littermates. Through this investigation, we serendipitously observed a population of peri-PLV cells that bind fluorescently-conjugated isotype control antibodies with the histologic appearance of mast cells (**Supplementary Figure 1**). Moreover, numbers of these proposed peri-PLV mast cells were markedly increased around the lymphatic vessels of TNF-tg mice. Based on these observations and the open questions about the role of peri-PLV mast cells on lymphatic function and association with RA pathogenesis, we hypothesized that: i) peri-PLV cells are indeed pro-inflammatory mast cells that regulate lymphatic function by releasing (degranulating) catabolic factors, and ii) genetic ablation and pharmacological inhibition of mast cells ameliorates lymphatic dysfunction and reduces inflammatory-erosive arthritis in TNF-tg mice.

## Materials and methods

### Mouse models

All animal research was conducted with approval by the University of Rochester Institutional Animal Care and Use Committee. TNF-tg mice (3647 line) (25) were initially acquired from Dr. George Kollias and have since been maintained at the University of Rochester. The TNF-tg mice were bred as heterozygotes, and WT littermates were used as controls (C57BL/6 genetic background). B6.Cg-*Kit^W-sh^*/HNihrJaeBsmJ (*Kit^W-sh/W-sh^* or cKit^-/-^) mice were purchased from The Jackson Laboratory (Bar Harbor, ME, Strain #:030764) and maintained at the University of Rochester vivarium. For crossing between the two strains, TNF-tg or TNF-tg x cKit^+/-^ mice were used as male breeders, and cKit^-/-^ mice as female breeders. For all *in vivo* longitudinal outcome measures, 4 to 5-month-old male mice were anesthetized with 1–3% isoflurane. All mice were euthanized with a lethal dose of ketamine/xylazine cocktail (intraperitoneal) followed by cervical dislocation. A total of 14 mice were used in this study, with a minimum of n = 3 experimental units evaluated for each group (n=8 for WT, n=4 for cKit^-/-^, n=4 for TNF-tg, n=3 for TNF-tg x cKit^-/-^). Out of the total 8 WT animals, 3 WT mice received all examinations. The remaining 5 WT mice underwent microCT and histologic assessment, as they served as controls for their same-age double transgenic mice, which unfortunately died before the dissection.

### Whole mount immunofluorescent microscopy

PLVs were harvested and fixed as previous described (4, 26). Briefly, fixed PLVs were blocked with 5% normal goat serum (NGS; ThermoFisher Scientific Cat# 50062Z)/1 × TBS/0.3% Triton X-100 (BioRad, Cat# 1706435) for 1 h at room temperature (RT), and then incubated with the primary antibodies diluted in 5% NGS/1 × TBS/0.3% Triton X-100 overnight at 4°C. For αSMA labeling, we utilized mouse anti-αSMA antibodies (AlexaFluor 488 conjugate; ThermoFisher Scientific Cat# 53-9760-82, 1:100 dilution). Primary conjugated isotype control staining was performed with mouse IgG2b antibodies (AlexaFluor 555; ThermoFisher Scientific Cat# A-21428, 1:100 dilution).

To validate the mast cell subtypes, the following primary antibodies were used in this study: Rabbit anti-human Mast Cell Tryptase IgG (mouse reactivity; Bioss Antibodies Cat# BSM-52533R; 1:100 dilution); Rat anti-mouse Mcpt-1 IgG1 (ThermoFisher Scientific Cat# 14-5503-80; 1:20 dilution); Goat anti-Mouse Mcpt-4 Polyclonal (ThermoFisher Scientific Cat# PA5-142454; 1:100 dilution); Rabbit anti-human CMA1(Mcpt-5) IgG (mouse reactivity; ThermoFisher Scientific Cat# PA5-79053; 1:50 dilution); Rabbit anti-mouse Chymase antibody (Abcam Cat# ab233103; 1:50 dilution). To prevent non-specific labeling of Fc receptors (FcR), exclusively secondary antibodies below featuring F(ab) fragments were utilized: Anti-rabbit IgG (H+L), F(ab’)2 Fragment (Alexa Fluor 555 Conjugate; Cell Signaling Technology Cat# 4413; 1:1000 dilution); F(ab’)_2_ Donkey anti-Rat IgG ((Alexa Fluor 647 Conjugate; Jackson ImmunoResearch Inc Cat#712-606-153; 1:1000 dilution); F(ab’)_2_ Rabbit anti-Goat IgG (Alexa Fluor 647 Conjugate; Jackson ImmunoResearch Inc Cat# 305-606-006; 1:500 dilution). After 3 × 10 min washes in 1 × TBS/0.1% Triton X-100 following antibody incubation, the PLVs were mounted on a microscope slide with one drop of both ProLong Gold Antifade Mountant (ThermoFisher Scientific Cat# P36930) and NucBlue Live ReadyProbes Reagent (Hoechst 33342 formulation; ThermoFisher Scientific Cat# R37605). The PLVs were then imaged using a VS120 Slide Scanner for αSMA coverage analysis as previously described (4, 26).

### Histochemistry

For histochemistry of mast cells, PLVs were subjected to two different preprocessing approaches for further toluidine blue and fast green (TBFG) staining. One group of PLVs was paraffin-embedded and longitudinally sectioned. Another group of PLVs had the coverslip carefully removed to enable whole mount TBFG staining after processing for WMIFM imaging in order to preserve the tissue at its original location and morphology for direct comparison. For sectioned PLVs, the sections underwent deparaffinization and hydration to distilled water, while the whole mounted PLVs were washed with distilled water. For TBFG staining, the tissue was exposed to 0.5% potassium permanganate for 2 minutes, followed by 1% potassium metabisulfite for 1 minute and rinsing with running tap water for 3 minutes. Subsequently, the tissue was stained with 0.02% toluidine blue for 5 minutes and 0.08% fast green for 30 seconds. Distilled water was used for a 30-second rinse between each staining procedure. Finally, the slides were dehydrated, mounted, and mast cells quantified.

For histochemistry of ankles and quantification of synovitis and osteoclasts, lower limbs of the mice were processed for paraffin embedded and demineralized hematoxylin and eosin (H&E; synovium) or tartrate-resistant acid phosphatase (TRAP; osteoclasts) histology, as previously described (9). All slides were scanned with an Olympus VS120 (full slide scans available upon request), and quantitative histomorphometry for synovial and TRAP^+^ tissue area was performed with Visiopharm (Hoersholm, Denmark, version 2021.04.0.10211), as previously described (4).

### Scanning electron microscopy

SEM was performed, as previously described (4). Briefly, PLVs were fixed in 4% paraformaldehyde/2.5% glutaraldehyde/0.1M cacodylate overnight, post-fixed in buffered 1% osmium tetroxide, dehydrated, critically point dried, mounted onto aluminum stubs and sputtercoated with gold prior to imaging using a Zeiss Auriga FE SEM. Three SEM micrographs per sample group were randomly chosen for descriptive analysis.

### Near infrared-indocyanine imaging and quantification of lymphatic function

The subcutaneous injection of 10 µl of a 0.1 mg/ml ICG solution in water was administered to both hind footpads of the mouse. The fluorescence of the footpad was measured in an IVIS Live Animal Imaging System (Caliper Life Sciences Inc.) one hour after injection (baseline) to capture the maximum fluorescence signal intensity, and again at six hours to quantify the remaining fluorescence, where the difference between these timepoints represents lymphatic clearance. Thus, the ratio of the fluorescence difference and baseline fluorescence was used to quantify lymphatic clearance as a biomarker of PLV function and drainage from the footpad.

### Micro-CT quantification of talus bone volume

Semiautomated quantification of talus bone volume was performed, as previously described (4). Briefly, micro-CT hindpaw datasets were acquired using a VivaCT 40 (Scanco Medical, Bassersdorf, Switzerland), and all *in vivo* scans were collected with the following imaging parameters: 55 kV, 145 μA, 300 ms integration time, 2048 × 2048 pixels, 1000 projections over 180°, resolution 17.5 μm isotropic voxels. Image collection for the hindpaws was completed in 30-45 min. Semiautomated 3D rendering of the ankle bones with volumetric quantification of the talus bone was performed with Amira software (ThermoFisher Scientific, version 4.6.0).

### Cromolyn sodium treatment

Ten 4-month-old female TNF-Tg mice received baseline micro-CT and near-infrared indocyanine green scans to determine ankle bone volume and lymphatic clearance, respectively, and randomized to 3-weeks of daily intraperitoneal injections of placebo (PBS) or cromolyn sodium (CS) (3.15 mg/kg), as previously described (27). All mice were included in the study, except for one CS-treated animal, which was excluded from histologic assessment due to post-measurement mortality. Otherwise, no mice or samples were excluded from the study.

### Bioinformatic single-cell RNA-sequencing analysis

To evaluate PLV-associated mast cells and other mast cell subtypes, we performed further bioinformatic analysis using our published single-cell RNA-sequencing (scRNAseq) dataset of PLVs (GSE190999,(28)) compared to a publicly available single-cell transcriptomics *Mouse Cell Atlas* (29–31). Analysis was performed in R (v4.1.2) with clustering and differential gene expression analysis performed in Seurat (v4.3.0), as previously described (28). The differentially expressed genes (log_2_ fold-change < or >1, FDR<0.05) were then input into WikiPathway and Elsevier pathway analyses.

### Statistics

We performed statistical analyses using GraphPad Prism (v9.1.1; Boston, MA, USA) to assess the significance of the observed differences. All statistical tests were two-tailed, and a p-value of less than 0.05 was considered statistically significant. Unpaired t-test was used in quantification of the MCT^+^ cells and FcR^high^ peri-PLV cells between WT and TNF-tg animals; and all comparison of WT, cKit^-/-^, TNF-tg and TNF-tg x cKit^-/-^ animals; and all comparison between CS treated or saline treated TNF-tg animals. Additionally, we employed linear regression analysis to examine inter-user reliability of determining non-degranulating mast cells vs degranulating mast cells, regression of ICG clearance % vs. % degranulating mast cells in TNF-tg mice, and the association between of % degranulating mast cells vs. age. The results were reported as simple linear regressions with R^2^ and p-value.

## Results

### Demonstration of increased peri-PLV mast cells in TNF-Tg mice with inflammatory arthritis

**Figure 1:**
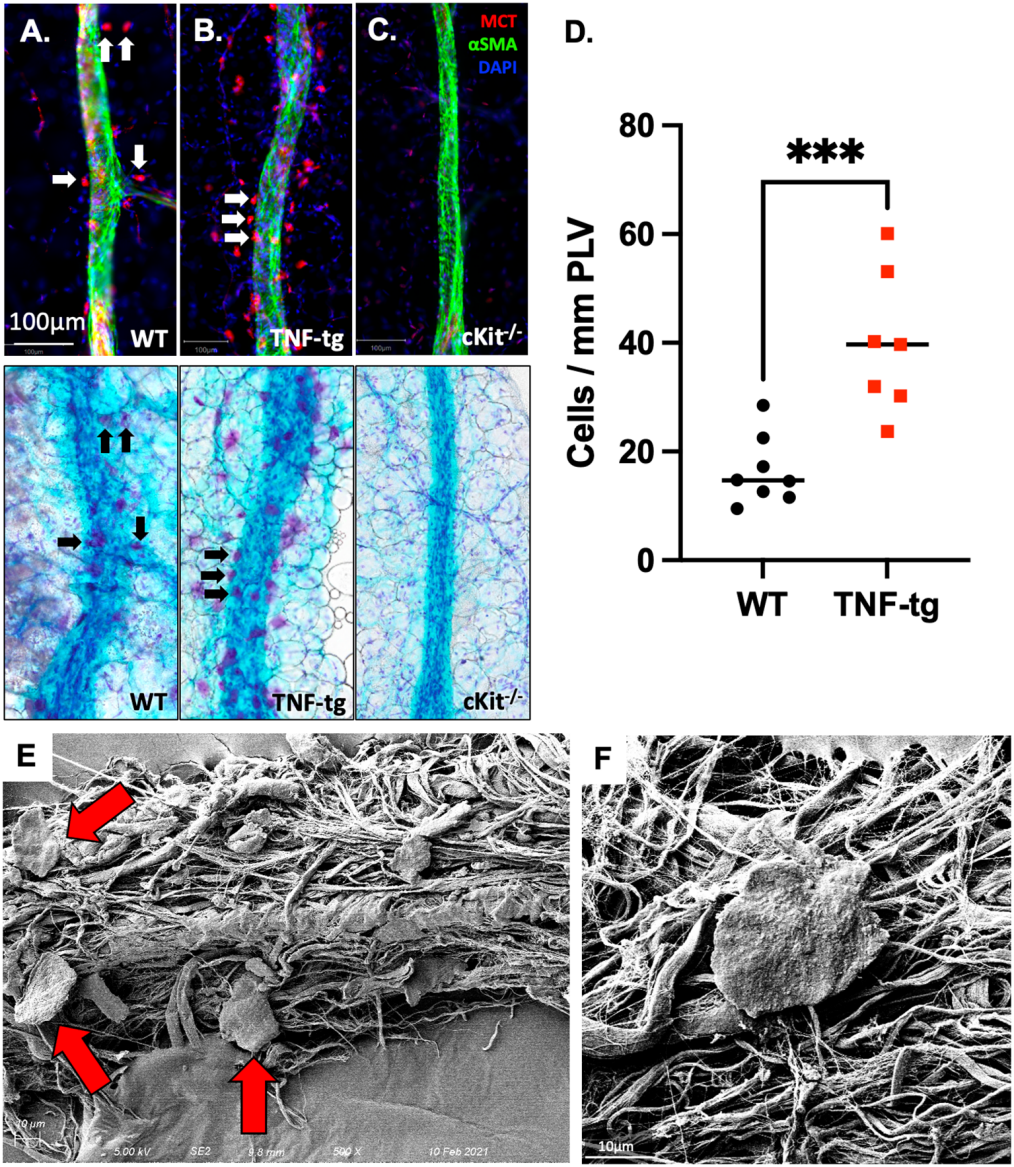
Mast cells accumulate around PLVs in TNF-tg mice with inflammatory arthritis. PLVs were harvested and processed for WMIF microscopy at 20x for αSMA (green) and mast cell tryptase (MCT, red). Subsequently, the same tissue underwent TBFG staining for light microscopy. Representative images are shown for WT **(A)**, TNF-tg **(B)**, and cKit^-/-^ **(C)** PLV. Note that the MCT^+^ cells (white arrows) are also toluidine blue stained (black arrows). The number of peri-lymphatic MCT^+^ cells per mm of WT and TNF-Tg PLV (n=8) was quantified, and data for each PLN are presented with the mean ± SD (**D**, ***p < 0.001 via unpaired t-test). Scanning electron microscopy (SEM) was performed on WT PLVs with attached peri-lymphatic tissue, and representative images obtained at x500 **(E)** and x5,000 **(F)** are shown highlighting mast cells with their typical pancake-shape (red arrows).

To verify the identity of the peri-PLV FcR^high^ population as mast cells, we performed WMIFM on PLVs from WT, TNF-tg, and mast cell deficient cKit-/- mice with primary antibodies against mast cell tryptase (MCT) and fluorescent secondary Fab, and then reimaged the PLV after histochemical staining for TBFG (**Figures 1A–C**). Consistent with our hypothesis, we observed MCT^+^ cells that also stained purple from the TBFG (indicating the presence of heparin and histamine within their granules (32)) around WT PLVs whose numbers were 2.8-fold increased around TNF-tg PLV (**Figure 1D**; WT: 16.41+/-6.269; TNF-tg: 39.86+/-12.93; p=0.0005), and were completely absent in tissue from cKit^-/-^ mice. To further validate the identity of these peri-PLV cells, we performed scanning electron microscopy, which demonstrated phenotypic pancake-shaped mast cells that were distributed around the connective tissue surrounding the PLV (**Figures 1E, F**, red arrows). Based on these results, we conclude that the expanded peri-PLV cells in TNF-tg mice are indeed mast cells.

### Degranulating peri-PLV mast cells negatively correlate with PLV function in WT and TNF-tg mice

**Figure 2:**
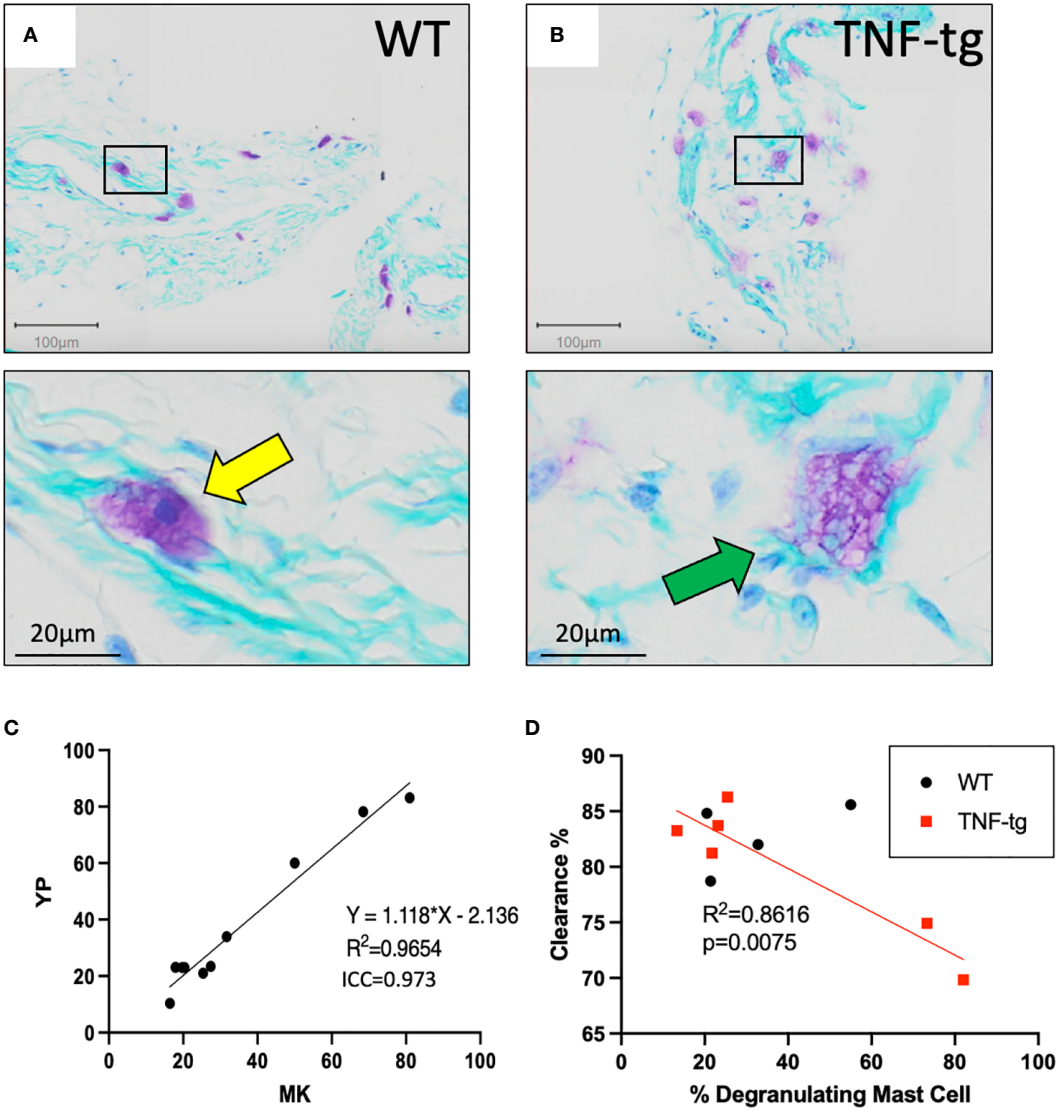
Degranulating peri-PLV mast cells are negatively associated with lymphatic function in WT and TNF-Tg mice. WT **(A)** and TNF-Tg **(B)** PLVs were processed for TBFG stained histology, and representative 5x images with corresponding 30x magnifications are shown to illustrate the non-degranulating mast cells (yellow arrow) with smaller and less obvious vacuoles in WT PLV, versus the degranulating mast cells with large granules (green arrow) associated with TNF-Tg PLV. **(C)** Histomorphometry by two observers (YP and MK) was performed to determine the ratio of degranulating: non- degranulating mast cells per PLV, and the data are presented as a linear regression with the slope, R^2^, and ICC values. **(D)** *In vivo* NIR-ICG imaging was performed on the lower limbs of WT and TNF-Tg mice to determine lymphatic clearance efficiency, and the imaged PLVs (n=6 for TNF-tg, n=4 for WT) were subsequently harvested to quantify the percentage of degranulating peri-PLV mast cells via histomorphometry of TBFG stained sections. The data are presented as simple linear regressions of ICG clearance % vs. % degranulating mast cells with p-value.

Scrutiny of the TBFG-stained PLV histology sections revealed that WT mast cells appear to be stable (**Figure 2A**), while most TNF-tg mast cells appeared to be degranulated with unclear cellular borders and large cytoplasmic vesicles (**Figure 2B**). To validate these distinct non-degranulating vs. degranulating mast cell phenotypes, two independent observers (YP & HMK) performed double-blinded histomorphometric analysis, which produced excellent inter-user reliability results (**Figure 2C**; R^2 ^= 0.9654, p < 0.0001, ICC = 0.973). Given that mast cells are known to release histamine and proinflammatory cytokines that impact the contractility of lymphatic muscle cells (LMCs) (21, 23), we hypothesized that release of proinflammatory mediators by peri-PLV mast cells via degranulation may contribute to PLV dysfunction during the progress of inflammatory-erosive arthritis. To test this, we quantified PLV draining function in both WT and TNF-tg mice using near-infrared indocyanine green imaging, after which we harvested the PLVs to quantify mast cell degranulation via TBFG stained histomorphometry. As predicted, we observed a robust negative correlation between the percentage of degranulating peri-vascular mast cells and the ICG clearance function of PLVs (**Figure 2D**; R^2 ^= 0.6711; p=0.0461). Of note is that the percentage of degranulating peri-PLV mast cells was not associated with age of the TNF-tg mice (**Supplementary Figure 2**), suggesting that this mast cell phenotype is primarily the result of TNF exposure in these mice independent of the chronicity of the inflammation. Together, these results demonstrate that TNF-tg mice exhibit both increased number and degranulation of peri-PLV mast cells, potentially releasing histamine and other effector molecules that might impact joint-draining lymphatic function.

### Mast cell deficiency (cKit^-/-^) leads to reduced lymphatic function and exacerbation of inflammatory-erosive arthritis in TNF-tg mice

**Figure 3:**
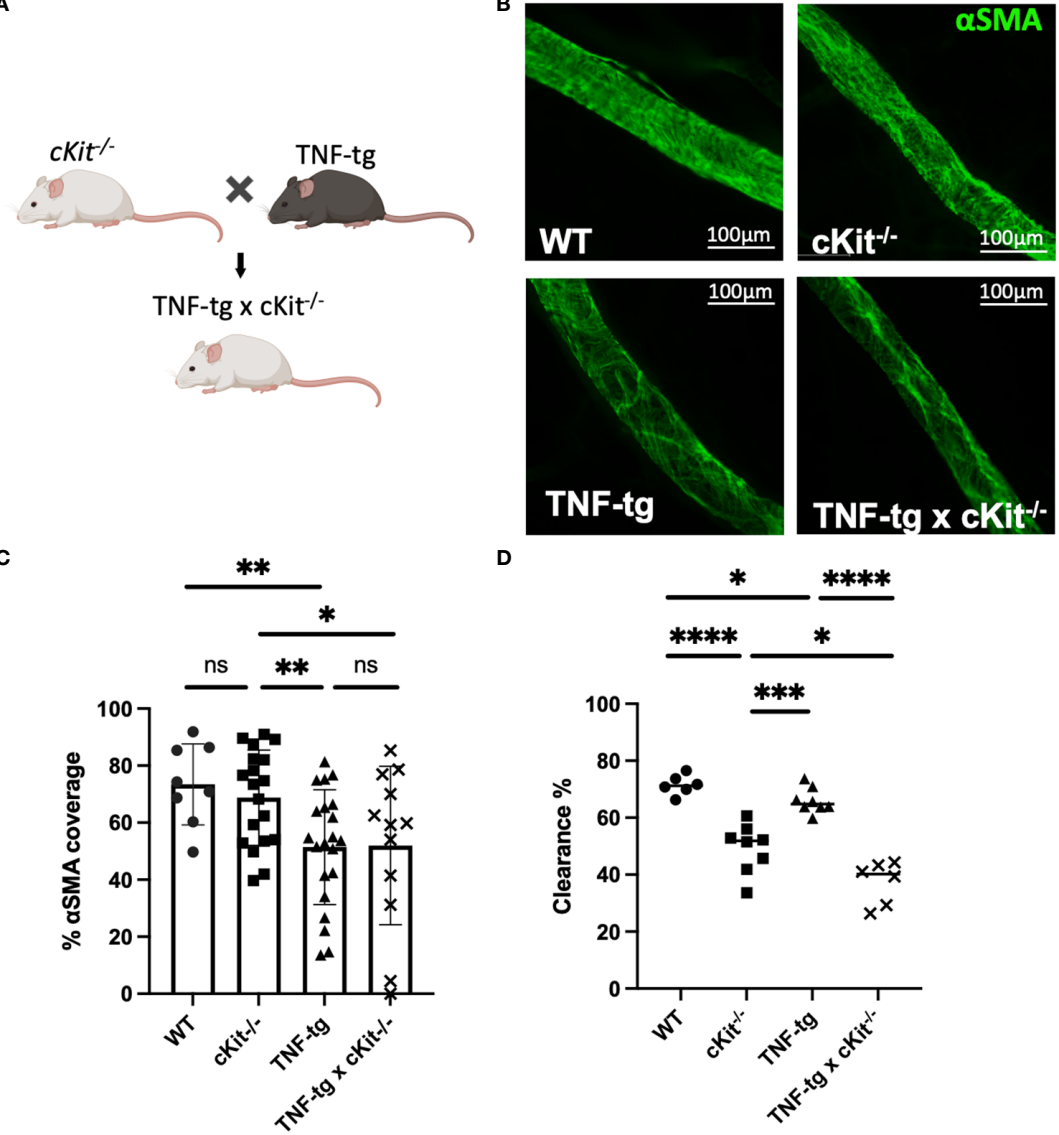
Mast cell deficiency does not affect αSMA coverage of PLVs. **(A)** B6.Cg-Kit^W- sh^/HNihrJaeBsmJ (cKit^-/-^) mice in a C57BL/6J background that have white fur were crossed with TNF-Tg mice in a C57BL/6J background that have black fur to generate TNF-tg x cKit^-/-^ mice that are runted and have white fur Schematic was created on BioRender.com. **(B)** PLVs were harvested from 4-month-old male and female mice for WMIF as in **Figure 1**, and representative x20 images of αSMA^+^ PLV-LMC coverage (green) are shown. **(C)** The % αSMA^+^ PLV-LMC coverage was quantified and the data for each PLV is shown with the mean for the group ± SD. **(D)** Quantification of lymphatic ICG clearance was performed as in **Figure 2** and the data for each lower limb are presented with the mean for the group (Unpaired t-test,*p<0.05, **p<0.01, ***p<0.001, **** p<0.0001).

To assess the roles of mast cells in lymphatic homeostasis and inflammatory-erosive arthritis, we performed WMIFM, near-infrared indocyanine green imaging, micro-CT and histomorphometry on WT, cKit^-/-^, TNF-tg and TNF-tg x cKit^-/-^ mice (**Figure 3A**). The WMIFM results showed that αSMA^+^ LMC coverage of cKit^-/-^ PLVs are similar to WT, in contrast to TNF-tg PLVs that are known to have decreased αSMA^+^ LMC coverage from chronic inflammation (4, 5). Moreover, mast cell deficiency did not further reduce αSMA^+^ LMC coverage of TNF-tg PLV (**Figures 3B, C**; p=0.9511). However, despite normal αSMA^+^ LMC coverage, cKit^-/-^ PLVs displayed greater functional defects in ICG clearance than TNF-tg PLVs, which presented as an additive functional deficiency in TNF-tg x cKit^-/-^ mice (**Figure 3D**; p<0.0001). Taken together, these findings imply that the mechanism underlying reduced lymphatic clearance in cKit^-/-^ mice is distinct from the loss of LMC structural proteins, and may in part be due to mast cell mediators known to influence lymphatic contractility (21, 23).

### TNF-tg mice exhibit exacerbated bone erosion with mast cell deficiency

**Figure 4:**
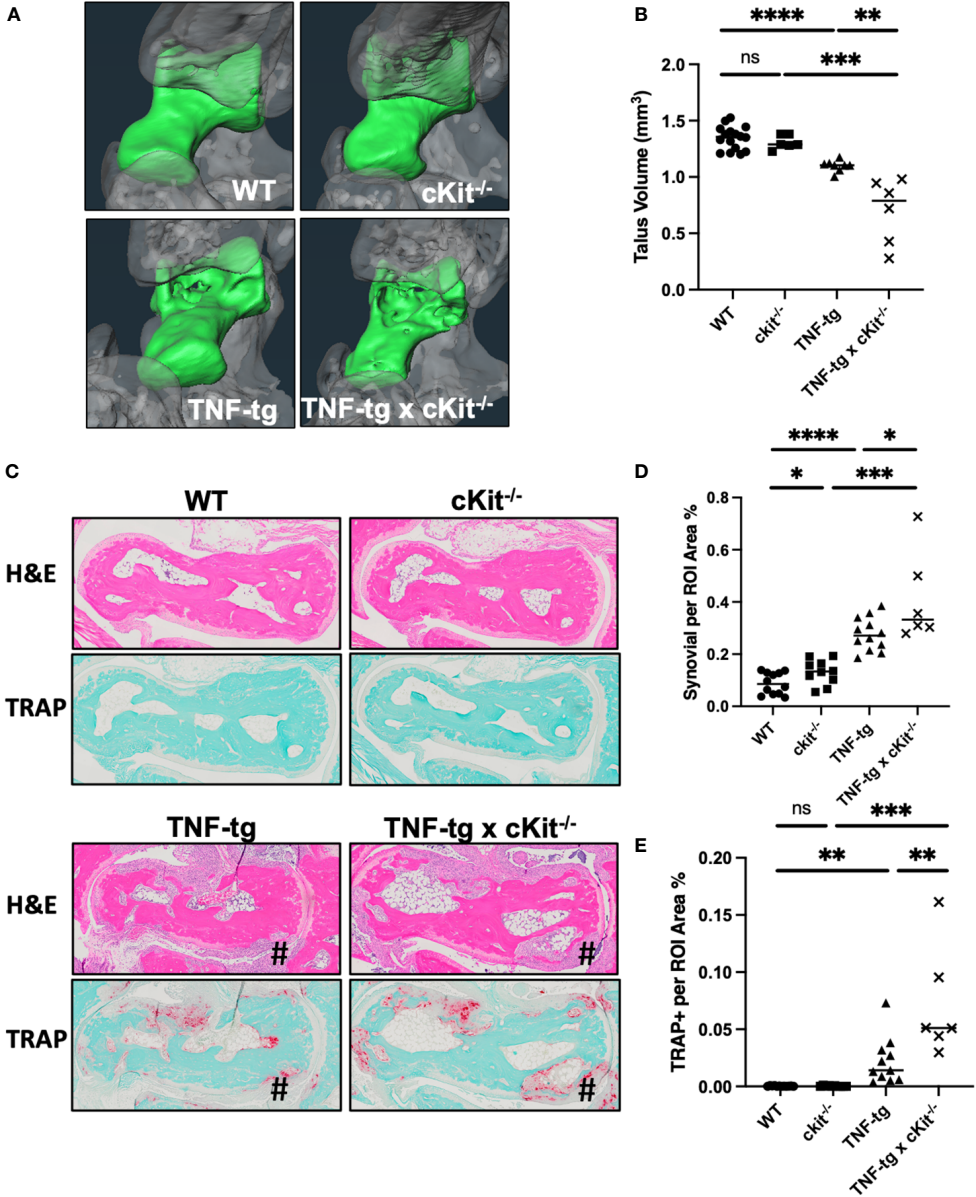
cKit deficiency exacerbates TNF-induced inflammatory-erosive arthritis in mice. **(A)** Micro-CT was performed on the ankles of the mice described in **Figure 3** and representative 3D renderings of the tali (green) are presented to illustrate the increased bone erosion in TNF-tg x cKit^-/-^ vs. TNF-tg mice. **(B)** Talus bone volume for each ankle are presented with the mean. **(C)** Representative H&E and TRAP-stained histology of the tali are shown at x4 to illustrate inflammation and osteoclasts at the site of bone erosion (#), with histomorphometry for synovitis **(D)** and osteoclasts **(E)**, and the data for each ankle (n ≥ 6) are presented with the mean (unpaired t test. *p<0.05, **p<0.01, ***p<0.001, **** p<0.0001).

Micro-CT and histological analyses of ankle bones failed to identify any differences between WT and cKit^-/-^ mice. The reduced bone volume, which is known to occur in TNF-tg mice (33, 34), is attributed to both synovitis and osteoclastic resorption. Synovial inflammatory tissue expresses osteoclast differentiation factors and is the source of osteoclasts in RA (35, 36). Osteoclasts mediate bone resorption inside-out from the subchondral bone and outside-in from the periarticular surface (37). The severity of synovitis, evaluated by the synovial volume in H&E staining (**Figures 4C, D**; p= 0.0259), and osteoclastic resorption, indicated by TRAP staining (**Figures 4C, E**; p= 0.0089), were significantly exacerbated in TNF-tg x cKit^-/-^, resulting in a greater reduction in bone volume compared to their TNF-tg littermates (**Figures 4A, B**; p= 0.0023).

Furthermore, mast cell deficiency was associated with reduced the overall health of TNF-tg mice as assessed by a significant decrease in body weight at ~4.5 month of age (**Supplementary Figures 3A, B**) and a significantly shortened life span (**Supplementary Figure 3C**) in both male and female TNF-tg x cKit^-/-^ mice.

### Pharmacological inhibition of mast cell degranulation exacerbates lymphatic drainage and inflammatory-erosive arthritis in TNF-tg mice

**Figure 5:**
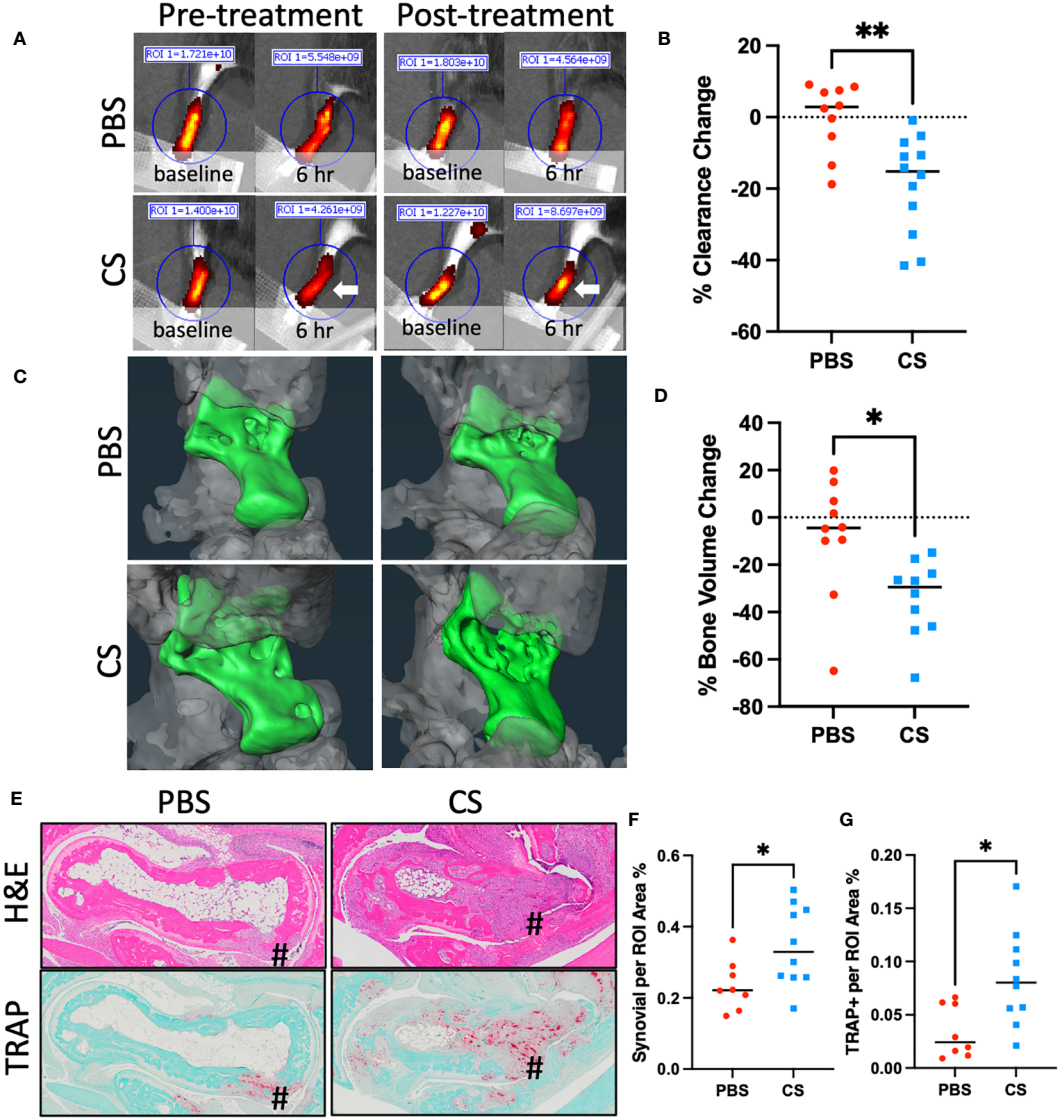
Cromolyn sodium treatment decreases lymphatic drainage and exacerbates inflammatory-erosive arthritis in TNF-Tg mice. 4-month-old female TNF-Tg mice (n = 5 animals; 10 lower limbs) were randomized to 3-weeks of placebo (PBS) or cromolyn sodium (CS), and longitudinal NIR-ICG imaging **(A, B)** and micro-CT **(C, D)** were performed to quantify lymphatic clearance and talus bone volume respectively. White arrow highlights the lack of clearance post-CS treatment. Post-treatment H&E and TRAP-stained histology are presented **(E)** with histomorphometry for synovitis **(F)** and osteoclasts **(G)** to illustrate exacerbated inflammatory-erosive arthritis (#) in CS treated TNF-Tg mice. Data for each lower limb (t-test, *p<0.05, **p<0.01).

As conclusions from our studies with TNF-tg x cKit^-/-^ mice are confounded by potential unaccounted defects during development and disruption of CD117/c-KIT and SCF/c-KIT signaling pathways in various organs (38), we aimed to corroborate our findings with pharmacological mast cell inhibition studies in adult TNF-tg mice with inflammatory-erosive arthritis. We selected cromolyn sodium (CS), an FDA-approved mast cell stabilizer that prevents release of inflammatory mediators such as histamine and leukotrienes, thereby inhibiting mast cell degranulation(39). In our study, two groups of 4-month-old female TNF-tg mice were treated with either CS or vehicle PBS for three weeks. The effectiveness of CS in inhibiting mast cell degranulation was confirmed by TBFG staining of PLV sections, which revealed a significantly decreased percentage of degranulating mast cells in the peri-PLV region after CS treatment, and markedly decreased overall health of TNF-tg mice (**Supplementary Figure 4**). Similar to cKit^-/-^ mice, our results showed that mice treated with CS exhibited reduced lymphatic clearance function compared to placebo controls (**Figures 5A, B**). Additionally, these mice showed increased bone erosion (**Figures 5C, D**) associated with more severe synovitis and osteoclast accumulation (**Figures 5E–G**).

These findings demonstrate the effectiveness of CS in inhibition of mast cell degranulation in the TNF-tg mouse model. Moreover, results from these pharmacology studies corroborate our genetic mast cell depletion results with cKit^-/-^ mice. Collectively, these findings demonstrate that mast cell activity (e.g. histamine release) is required for normal lymphatic function and that global inhibition of mast cells, either through genetic ablation or pharmacologic inhibition, exacerbates TNF-induced inflammatory-erosive arthritis in mice.

### Identification of a distinct population of intra-lymphatic vessel mast cells

**Figure 6:**
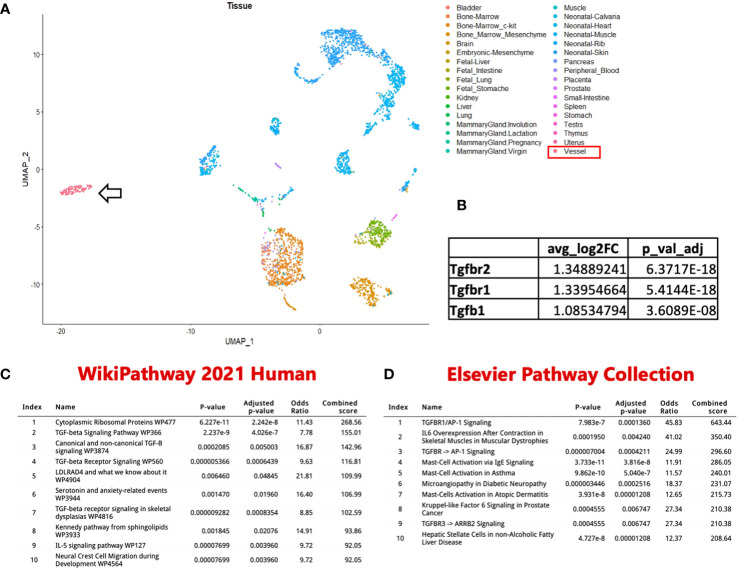
Identification of a unique PLV intravascular MC population. **(A)** In silico datasets were used to extract MC based on *Mcpt4, Cmal, Cpa3, Tpsb2, Kit, Fcerla & Gata2* expression from a comprehensive UMAP of 242k cells encompassing various organs in the Mouse Cell Atlas. This subset was then re-clustered and merged with PLV MCs from our previously published dataset. Notably, the PLV MCs (arrow) exhibited a distinct clustering pattern. **(B)** These PLV MCs have increased TGFβ signaling compared to the other MC clusters. ENRICHR Gene Ontology analysis identified enriched pathways and functional groups among genes with increased expression in the PLV MC cluster, with the ten most significant groups represented using WikiPathway 2021 human database **(C)** and Elsevier pathway collection database **(D)**. Enrichr table obtained from the Enrichr web resource (https://amp.pharm.mssm.edu/Enrichr), were utilized for the representation of these findings.

To better define the cellular subsets within and surrounding PLVs of WT and TNF-tg mice, we recently published a single-cell RNA-sequencing (scRNAseq) study describing altered peri-PLV immune and stromal cells in inflammatory-erosive arthritis (28). Although these experiments were designed to primarily focus on LMCs, we obtained data on various hematopoietic cells including mast cells, although in small numbers as live peri-PLV mast cells are difficult to isolate following stringent dissection, tissue digestion, and cell washing steps used to isolated individual LMCs. To better understand these cells that are physically associated with PLVs and express mast cell marker genes (*Mcpt4, Cma1, Cpa3, Tpsb2, Kit, Fcer1a & Gata2*), we completed an *in silico* bioinformatic study that combined these data with datasets from a comprehensive UMAP of 242,000 cells encompassing various organs in the *Mouse Cell Atlas* (29). The extraction was performed based on the expression of the mast cell marker genes, and following hierarchical re-clustering and integration, we derived a UMAP that revealed PLV mast cells that are phenotypically distinct from known mast cell populations in mice (**Figure 6A**). Interestingly, this cluster of PLV mast cells displayed increased TGF-beta signaling compared to other mast cell clusters via differential gene expression analysis (**Figure 6B**). To investigate the functional genomic implications of this discovery, we performed ENRICHR Gene Ontology analysis on the genes exhibiting significantly increased expression in the PLV mast cell cluster. This analysis allowed us to identify pathways that may be upregulated in these PLV mast cells versus other mast cell clusters. The results from pathway analyses conducted using the WikiPathway 2021 human database and the Elsevier pathway collection database are presented in **Figures 6C, D**, respectively. Notably, the PLV mast cell cluster displayed an elevated TGF-beta signaling pathway accompanied by increased mast cell activation, indicating potential anti-inflammatory and anabolic functions. While this preliminary *in silico* finding of increased TGF-beta signaling in WT peri-PLV mast cells is provocative, it is possible that signaling pathways in homeostatic mast cells are different from peri-PLV mast cells in TNF-tg mice with inflammatory arthritis. Thus, TGF-beta signaling might not fully explain the contribution of mast cells in this model.

**Figure 7:**
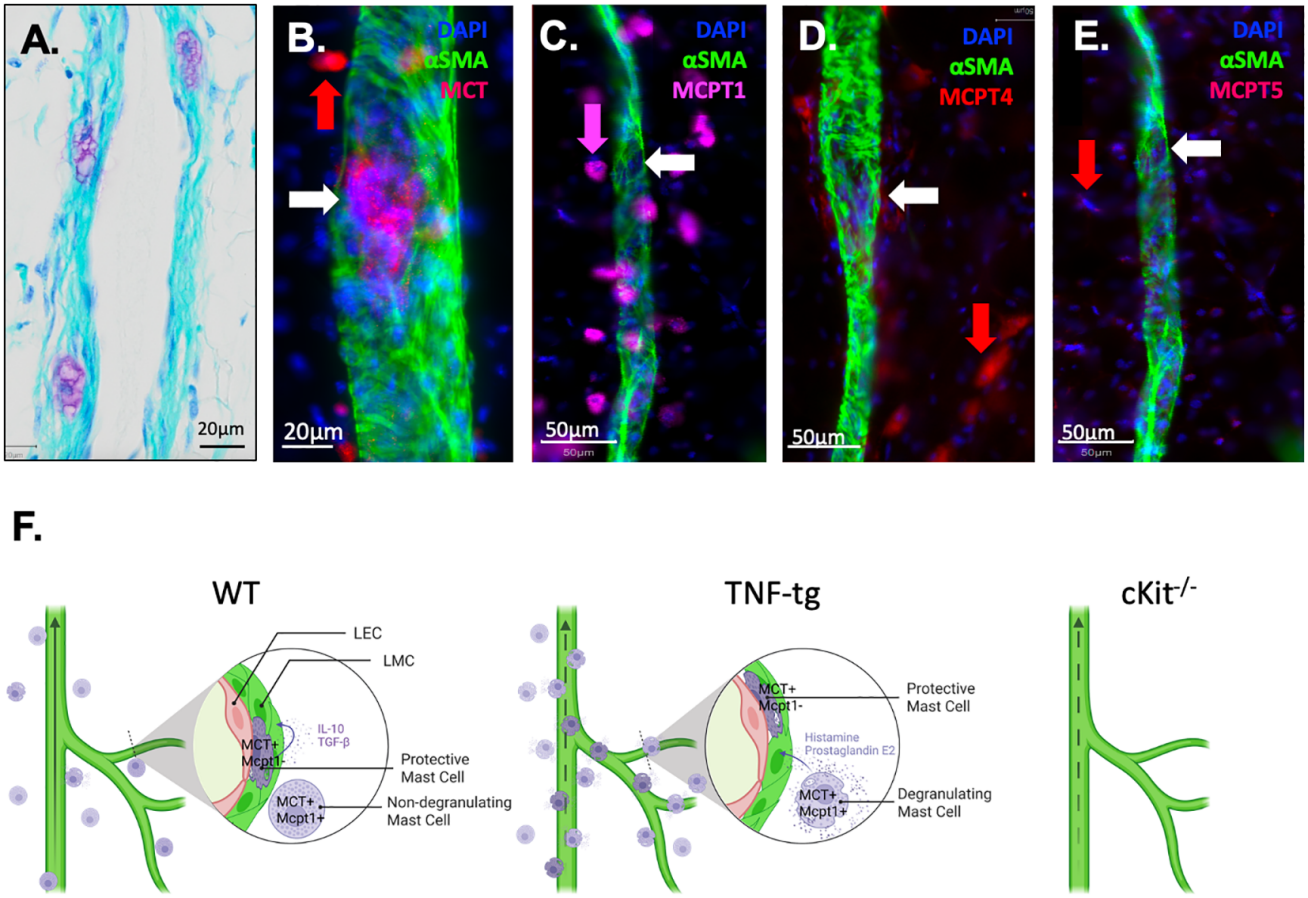
Phenotypic distinction between peri-PLV mast cells versus intravascular mast cells and proposed model of their pro and anti-inflammatory functions. **(A)** A 20x micrograph of a TBFG stained WT PLV is shown to highlight the novel intravascular MC identified in this study, which is embedded within the PLV wall, situated between the layers of lymphatic endothelial cells and lymphatic muscle cells. WMIFM was performed on TNF-tg PLV for αSMA and MCT, MCPT1, MCPT4 and MCPT5 as described in **Figure 1**. **(B)** 40x image highlighting MCT^+^ perivascular MC (red arrow) and MCT1^+^ intravascular MC (white arrow). **(C)** 20x image highlighting MCPT1^+^ perivascular MC (pink arrow) and MCPT1^-^ intravascular MC (white arrow). **(D)** 20x image highlighting MCPT4^+^ perivascular MC (red arrow) and MCPT4^-^ intravascular MC (white arrow). **(E)** 20x image highlighting MCPT5^-^ perivascular MC (red arrow) and MCPT5^-^ intravascular MC (white arrow). **(F)** Schematic illustration of MCT^+^/MCPT1^+^ conventional pro-inflammatory peri-lymphatic vessel mast cells and a novel homeostatic MCT^+^/MCPT1^-^ intra-lymphatic vessel mast cell population Schematic was created on biorender.com.

An unexplained finding in our prior αSMA WMIFM studies in lineage tracing and PLV repair experiments is the frequently observed presence of rounded cell-shape-like empty spaces within PLVs that lacked αSMA staining (4, 26, 40). Based on our *in silico* data predicting that a unique PLV-associated mast cell population exists, we re-examined our PLV histology and discovered purple toluidine blue stained cells buried within the lymphatic vessel wall (**Figure 7A**), located between the layers of lymphatic endothelial cells and LMCs. Given the similar size of these cells to the empty spaces in our αSMA WMIFM images, which we have repeatedly observed in our prior studies (4, 26, 40), we hypothesized that these “voids” might be occupied by a distinct population of intra-PLV mast cells. To test this, PLVs were subjected to WMIFM for αSMA and MCT, and we found that the gaps between the αSMA^+^ LMC contained cells with similar positive staining for MCT as the peri-PLV mast cells (**Figure 7B**), confirming the presence of intravascular mast cells within PLV. To further characterize these peri- and intra-PLV mast cells, we performed WMIFM with antibodies specific from mast cell proteases (MCPT1, 4 & 5). The results demonstrated that peri-PLV mast cells are MCPT1^+^ MCPT4^+^ while intra-PLV mast cells are MCPT1^-^ MCPT4^-^ (**Figures 7C, D**), further confirming that these are distinct populations of mast cells. Despite our prediction that these mast cells expressed MCPT5 from the scRNAseq data (**Supplementary Table 1**), our WMIFM studies failed to detect any positive staining in or around PLVs (**Figures 7E**).

## Discussion

Mast cells modulate the course of inflammatory arthritis through recruitment and activation of synovial macrophages (13), which produce the bulk of the pro-inflammatory cytokines in the RA joint, most notably TNF and IL-1. Of note is that mast cells also release these pro-inflammatory cytokines in inflammatory arthritis following JNK1-mediated mast cell degranulation (41). Additionally, clinical studies have shown that mast cells in early RA are associated with disease severity, and support B cell autoantibody production (18, 42).

Mast cells have also been studied in animal models of RA for over 50 years (43), and the results have varied based on the particular murine model used and experimental approaches. Some studies showed that mast cell depletion in the preclinical phase of collagen-induced arthritis (CIA) reduces the clinical outcome by lowering the inflammatory cytokine profile (44), while others demonstrated that CIA is not impaired in mast cell-deficient mice (45). In contrast, studies in the K/BxN serum-induced arthritis model showed that both W/Wv and Sl/Sld mast cell deficient mice are resistant to development of joint inflammation. Sl/Sld mice are only capable of encoding a soluble truncated cKit ligand that lacks both transmembrane and cytoplasmic domains(46, 47), while cKit ligand in W/Wv mice lacks kinase activity and is not expressed on the cell surface(48). Bone marrow mast cell engraftment restores susceptibility to arthritis in W/Wv mice, confirming that the resistance to arthritis induced by K/BxN serum transfer was due to the absence of mast cells in the mutant mice, and not to defects in other cell types (49).

Interestingly, Schubert et al. found a redundant role of mast cells in the K/BxN model, where joint inflammation was triggered by cartilage-bound immune complexes independently of T cells, while mast cells were critical for T-cell dependent CIA (50). Collectively, these studies suggest that mast cells may function as a cellular link between soluble mediators, autoantibodies, and other effector cells (i.e., T cells and osteoclasts), but appear to be dispensable for antibody-induced arthritis.

While the aforementioned work was largely focused on mast cells in the RA joint and animal models of acute inflammatory-erosive arthritis, our research interests have been centered on questions of arthritic flare in the setting of long-standing RA and progressive joint disease in chronic models. This unbiased research uncovered several parallel changes that occur in joint draining lymphatic vessels and lymph nodes of RA patients and TNF-tg mice, and have been broadly characterized by an initial “expansion” phase of mild disease with lymphangiogenesis and enlargement of efferent lymph nodes, followed by loss of efferent lymphatics, “collapse” of the joint draining lymph nodes and exacerbated synovitis and joint erosion at end-stage disease (8, 51). As the lymphatic deficits during the collapse phase are intrinsic to the lymphatic vessels themselves (3, 5), we completed a scRNAseq analysis of PLV from TNF-tg mice with inflammatory-erosive arthritis vs. WT controls (28). While this study confirmed our hypothesis of lymphatic muscle cell and M2 macrophage loss with a commensurate increase in inflammatory monocytes in TNF-tg lymphatic vessels, we also observed a surprising 3.6-fold decrease in the number TNF-tg mast cells, in addition to other hematopoietic cells that may also affects on lymphatic function. This observation also appears to be inconsistent with our finding of a 2.6-fold increase in peri-PLV mast cells in TNF-tg vs. WT observed using WIFM for MCT (**Figure 1**). However, it is important to note that the stringent PLV dissection, enzymatic digestion, and single-cell isolation procedures used for scRNAseq would be expected to eliminate the loosely adherent and unstable-degranulating mast cells observed in **Figure 1** from the analysis. Thus, we interpret this loss of mast cells in TNF-tg PLV to be a specific decrease in the novel intravascular mast cells which exist stably embedded within vessel walls that we identified in this study (**Figures 7A, B**).

Based on the literature and our novel findings, we conclude that subpopulations of mast cells exist in and around lymphatic vessels (**Figure 7**). During homeostasis, the peri-lymphatic MC are dormant in their non-degranulating form, while the intravascular MC release anti-inflammatory and anabolic factors that sustain lymphatic vessel structure and function. Thus, global loss of intravascular MC in cKit^-/-^ results in loss of lymphatic drainage. Chronic inflammatory arthritis stimulates accumulation and degranulation of peri-vascular MC, which produce pro-inflammatory and catabolic factors that overwhelm intravascular MC to permeabilize and degenerate lymphatic vessels. As other cells also produce these factors during chronic inflammation, loss of all MC including the intravascular MC in cKit^-/-^ and cromolyn sodium treatment has a net result of exacerbated synovitis and erosive arthritis (**Figure 7F**).

We hypothesize that the TNF-induced peri-PLV mast cells are of the canonical pro-inflammatory subtype, while the mast cells embedded within the vessel are of a homeostatic/anti-inflammatory subtype that has a protective role during inflammatory arthritis. Our *in silico* data demonstrating potential TGF-beta signaling in these intra-PLV mast cells suggest that they may be akin to mast cells involved in wound healing (52). It is also interesting that these PLV-associated mast cell populations are phenotypically distinct based on MCPT1 and MCPT4 expression (**Figure 7C**), which opens possibilities for selective-inducible depletion of a specific mast cell population with genetic models. It might also be possible to isolate mast in the presence of mast stabilizers, and assess functional characteristics between WT vs TNF-tg mast cells *in vitro*.

Several noteworthy limitations to our study deserve mention. Most notable is that our experimental approach to assess MC loss of function (cKit^-/-^ and CS treated mice) affects all MC systemically, such that specific conclusions of the distinct roles of peri-PLV and intra-PLV MC cannot be made at this time. Nonetheless, our hypothesis generating findings support our speculation that intravascular mast cells play a homeostatic/protective role in lymphatic function and joint preservation is based on the simplest explanation of the observed defective ICG clearance in cKit^-/-^ and CS treated mice, and exacerbated inflammatory-erosive arthritis in mast cell deficient TNF-tg mice, and warrants future studies to confirm this with direct evidence. Additional support for this speculation also came from functional genomic data demonstrating increased TGF-beta signaling in intravascular mast cells compared to other mast cell subtypes. However, our attempts to confirm this via WMIFM of TGF-beta and TGF-beta receptors failed to produce credible data, and efforts to improve our methods are ongoing.

Another limitation relates to the *Kit^W-sh/W-sh^* mouse model, which exhibits mast cell deficiency and maintains normal levels of differentiated hematopoietic and lymphoid cells without anemia or sterility, making it a suitable choice for our study considering its favorable characteristics compared to *Kit^W/W-v^* mice. However, *Kit^W-sh/W-sh^* mice still display c-Kit tyrosine kinase-dependent signaling related abnormalities, lack interstitial cells of Cajal in the gut, and exhibited bile reflux into the stomach. Although it is unlikely that these defects contribute to the specific PLV and ankle arthritis phenotypes we observed, we acknowledge these confounders in our data analyses, and the importance of investigating mast cell subtypes and selectively inhibiting or deleting inflammatory mast cells. Based on our finding of differential expression of MCPT1 and MCPT4 in peri vs. intravascular mast cells, this should be possible using a genetic approach that utilizes *Mcpt1-*target *or Mcpt4*-target gene expression akin to Mcpt1-Cre-iDTR mice (53). Our future research will focus on generating MC-subpopulation-specific mast cell strains, such as the Mcpt1 DTR murine model, which would enable selective *in vivo* depletion of Mcpt1-expressing mast cells (54). Alternatively, it might be possible to generate a depletion model with CRISPR to selectively remove the identified mast cell subpopulations *in vitro* in the presence of mast cell stabilizer.

Our *in silico* analysis revealed the expression of Mcpt5, which is a mast cell chymase typically found in connective tissue mast cells (55). However, despite our efforts, we were unable to detect any Mcpt5^+^ mast cells via WMIFM. This could be attributed to post-transcriptional regulation of Mcpt5 or potential issues with the antibodies used in our study. Thus, future studies are needed to sort this out.

In summary, we demonstrate that two distinct populations of mast cells exist in and around joint draining PLV. The peri-PLV mast cell population expands in the setting of chronic TNF-induced inflammatory-erosive arthritis, and likely contributes to lymphatic dysfunction as their degranulated fraction is inversely correlated with ICG clearance. However, as genetic depletion and pharmacologic inhibition of mast cells exacerbates inflammatory-erosive arthritis and lymphatic dysfunction without damaging LMCs, we infer that intra-PLV mast cells may produce critical signals for LMC contraction and efficient lymph flow, and future studies to test this are warranted. Additionally, as prior mast cell loss of function studies in arthritis models have focused on direct effects within the joint, interpretation of these results may benefit from consideration of the mast cell indirect effects on arthritis via their regulation of lymphatic function.

## Data availability statement

The datasets presented in this study can be found in online repositories. The names of the repository/repositories and accession number(s) can be found in the article/**Supplementary Material**.

## Ethics statement

The animal study was approved by University of Rochester Committee for Animal Resources. The study was conducted in accordance with the local legislation and institutional requirements.

## Author contributions

YP: Writing – original draft, Writing – review & editing. HK: Writing – review & editing. KB: Writing – review & editing. LX: Writing – review & editing. CR: Writing – review & editing. ES: Funding acquisition, Project administration, Supervision, Writing – review & editing.
