# Supplementary figures and images for: Distinct mast cell subpopulations within and around lymphatic vessels regulate lymph flow and progression of inflammatory-erosive arthritis in TNF-transgenic mice
Source: Front Immunol. 2023 Dec 14;14:1275871. doi: 10.3389/fimmu.2023.1275871 (PMC10752982; doi:10.3389/fimmu.2023.1275871)

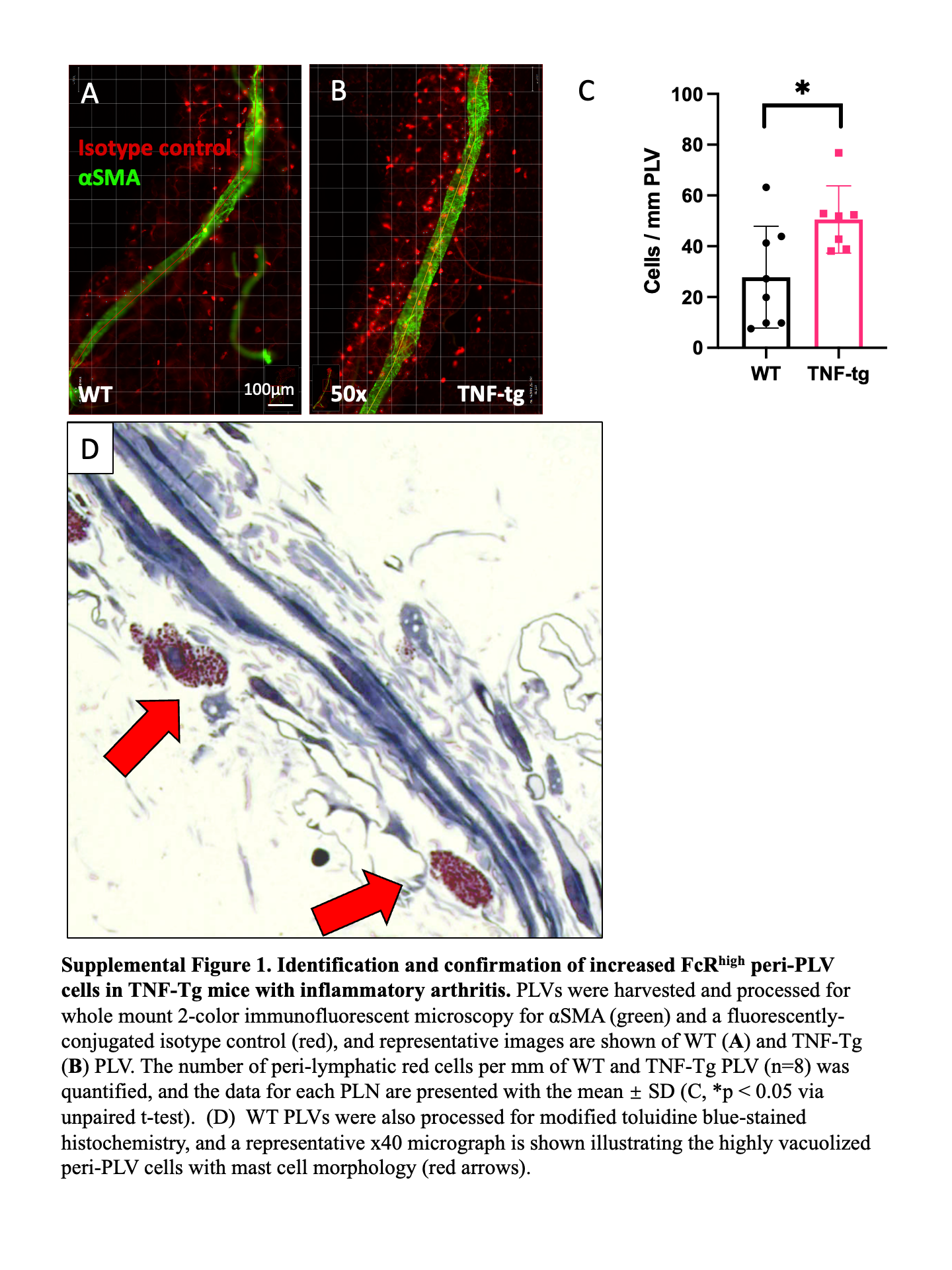

Supplement: Supplementary file 1 [file Image_1.tiff]

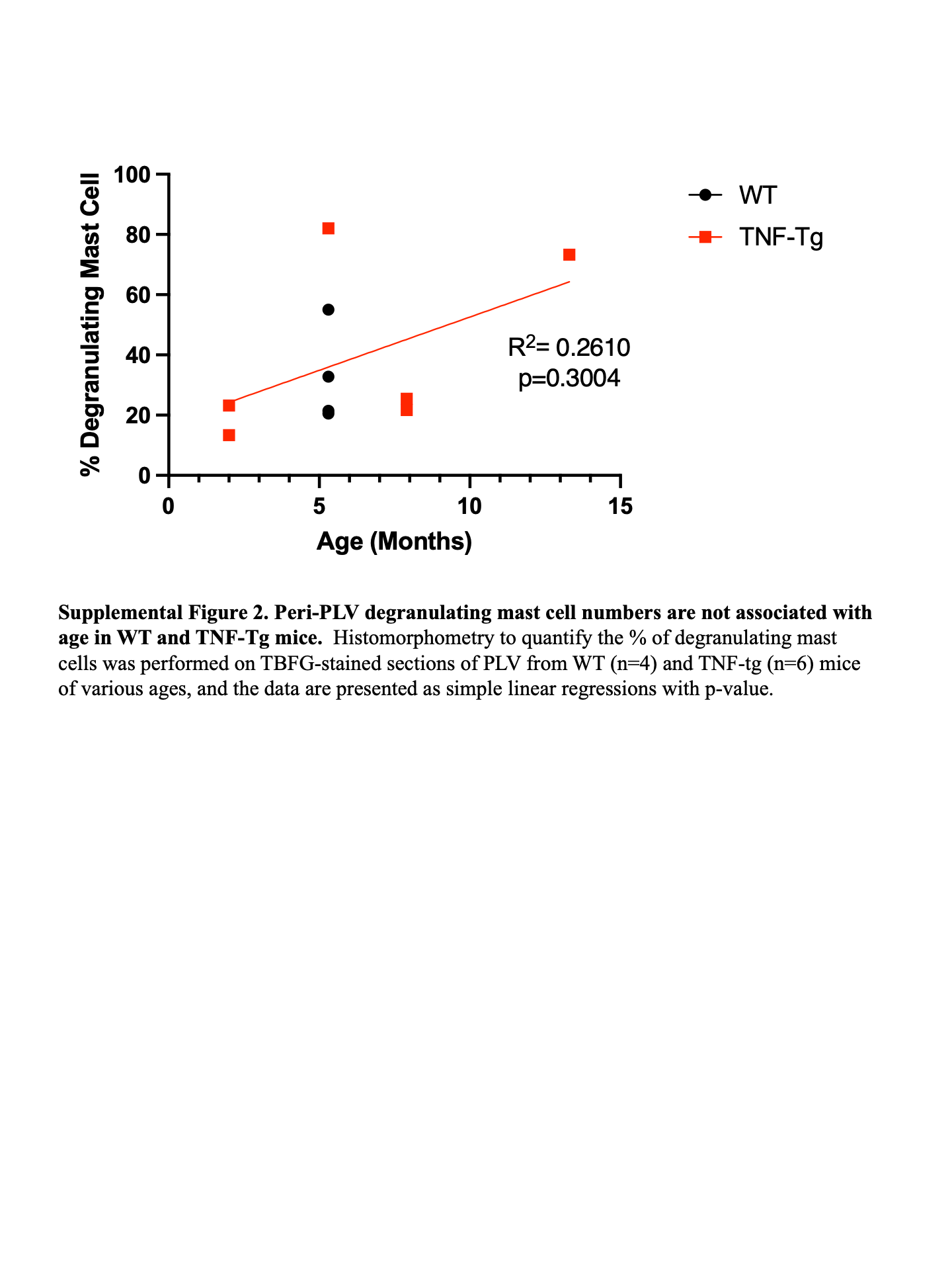

Supplement: Supplementary file 2 [file Image_2.tiff]

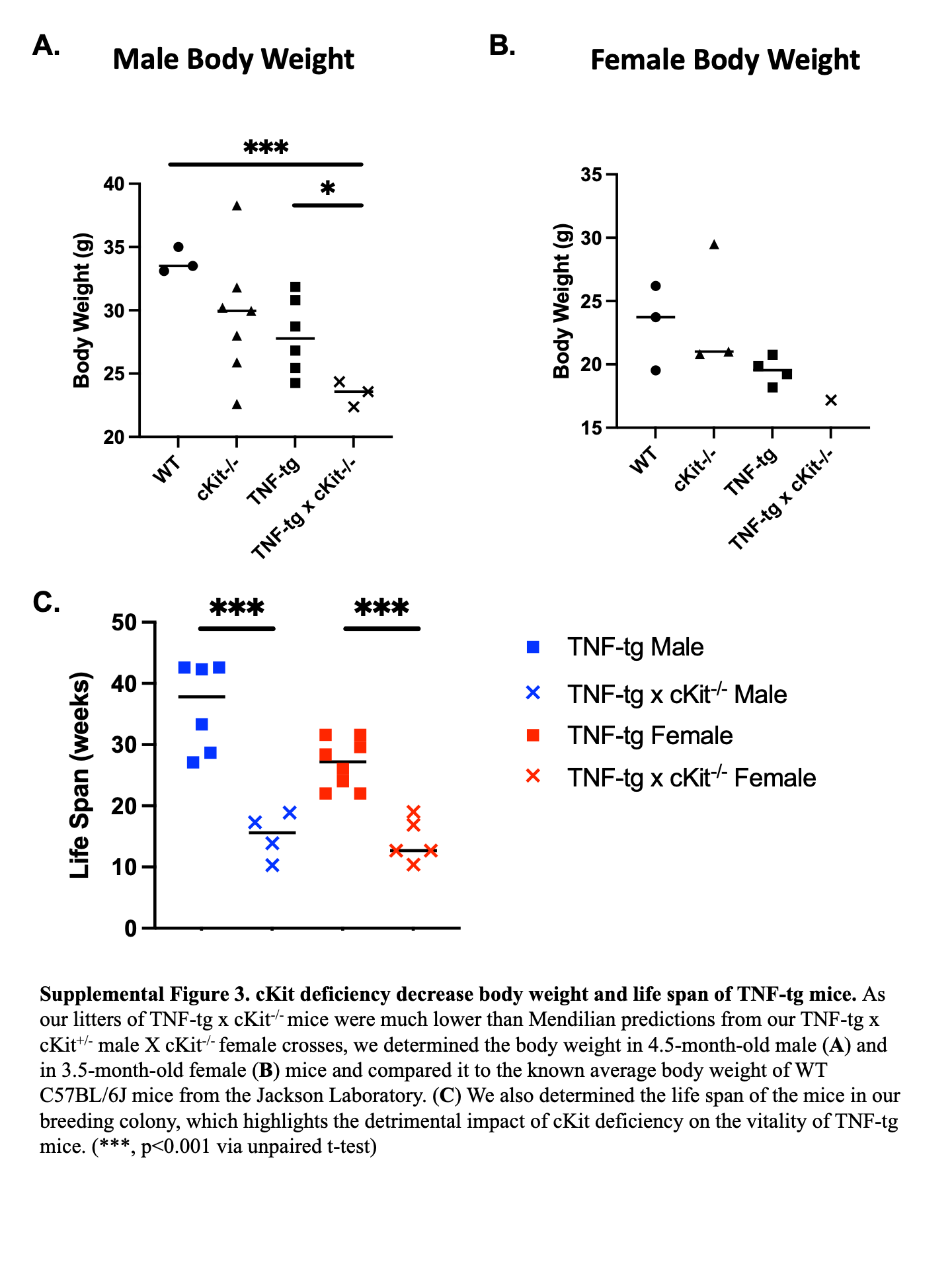

Supplement: Supplementary file 3 [file Image_3.tiff]

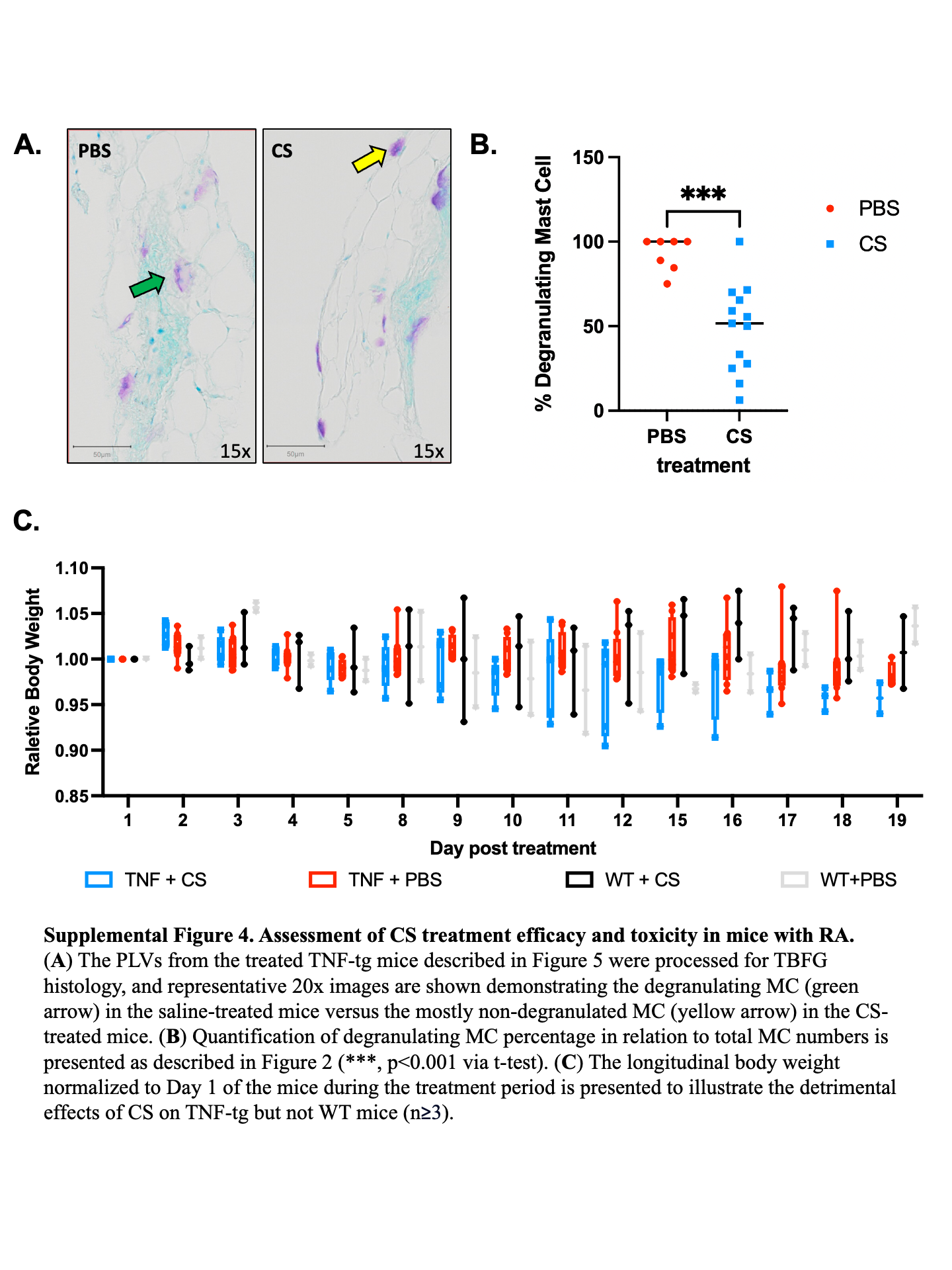

Supplement: Supplementary file 4 [file Image_4.tiff]
